# Impact of COVID-19 pandemic on the prevalence of respiratory viruses in children with lower respiratory tract infections in China

**DOI:** 10.1186/s12985-021-01627-8

**Published:** 2021-08-03

**Authors:** Pengcheng Liu, Menghua Xu, Lingfeng Cao, Liyun Su, Lijuan Lu, Niuniu Dong, Ran Jia, Xunhua Zhu, Jin Xu

**Affiliations:** Department of Clinical Laboratory, Children’s Hospital of Fudan University, National Children’s Medical Center, 399 Wanyuan Rd, Minhang District, Shanghai, 201102 China

**Keywords:** COVID-19, Nonpharmaceutical interventions, Respiratory viruses, Prevalence, Children

## Abstract

**Background:**

The multifaceted non-pharmaceutical interventions (NPIs) taken during the COVID-19 pandemic not only decrease the spreading of the SARS-CoV-2, but have impact on the prevalence of other viruses. This study aimed to explore the prevalence of common respiratory viruses among hospitalized children with lower respiratory tract infections (LRTI) in China during the COVID-19 pandemic.

**Methods:**

Respiratory specimens were obtained from children with LRTI at Children’s Hospital of Fudan University for detection of respiratory syncytial virus (RSV), adenovirus (ADV), parainfluenza virus (PIV) 1 to 3, influenza virus A (FluA), influenza virus B (FluB), human metapneumovirus (MPV) and rhinovirus (RV). The data were analyzed and compared between the year of 2020 (COVID-19 pandemic) and 2019 (before COVID-19 pandemic).

**Results:**

A total of 7107 patients were enrolled, including 4600 patients in 2019 and 2507 patients in 2020. Compared with 2019, we observed an unprecedented reduction of RSV, ADV, FluA, FluB, and MPV infections in 2020, despite of reopening of schools in June, 2020. However, the RV infection was significantly increased in 2020 and a sharp increase was observed especially after reopening of schools. Besides, the PIV infection showed resurgent characteristic after September of 2020. The mixed infections were significantly less frequent in 2020 compared with the year of 2019.

**Conclusions:**

The NPIs during the COVID-19 pandemic have great impact on the prevalence of common respiratory viruses in China. Meanwhile, we do need to be cautious of a possible resurgence of some respiratory viruses as the COVID-19 restrictions are relaxed.

## Introduction

An outbreak of the coronavirus disease 2019 (COVID-19) caused by severe acute respiratory syndrome coronavirus 2 (SARS-CoV-2) has emerged in Wuhan, Hubei province, China, in December 2019 [1]. This outbreak then grew substantially and the World Health Organization (WHO) declared COVID-19 to be a public health emergency of international concern (PHEIC) on January 31, 2020 [2].

To control the pandemic of COVID-19, China has taken a series of non-pharmaceutical interventions (NPIs) against COVID-19 in accordance with state instructions, such as prohibiting social gatherings, wearing masks and encouraging employees to work at home. The Chinese government also suggested postponing the spring 2020 semester in primary and middle schools. Consequently, all face-to-face teaching in schools has been cancelled, and students must receive online courses at home during the COVID-19 pandemic, until the schools reopen in early June, 2020 [3]. No previous NPIs can be compared to the extent of the lockdown implemented during the COVID-19 outbreak. These multifaceted NPIs not only decrease the spreading of the COVID-19, but were associated with a significant decrease in infectious diseases disseminated through airborne or fecal–oral transmission, such as common cold, seasonal influenza, bronchiolitis, gastroenteritis, and acute otitis [4–6].

Lower respiratory tract infections (LRTI), such as pneumonia and bronchiolitis, are a substantial public health problem and a leading cause of morbidity and mortality among children younger than 5 years [7, 8]. The aetiology of LRTI is diverse and complicated, which may vary according to gender, age, and season [9]. Respiratory viruses play an important role in LRTI. A previous multi-site, international case–control study showed that viruses, especially RSV, were the predominant cause of pneumonia requiring hospital admission in children younger than 5 years [10]. However, it is unclear how the viral etiology and clinical epidemiologic features changed among the children hospitalized for LRTI during the COVID-19 pandemic in China.

The aim of this study is to report and discuss the impact of the COVID-19 pandemic (the year of 2020) on the prevalence of common respiratory viruses among hospitalized children with LRTI in Shanghai, China, and to compare it with prevalence patterns in the year of 2019 (before COVID-19 pandemic).

## Methods

### Study design

To explore the changes in epidemiology of viral etiology in children with LRTI during the COVID-19 pandemic, the laboratory results and clinical data of children admitted to Children’s Hospital of Fudan University with LRTI in the entire year 2020 (COVID-19 pandemic) were analyzed. Data were then compared with the same date ranges in 2019.

The inclusion criteria for this study were: (1) children aged younger than 18 years; (2) hospitalization at the Children’s Hospital of Fudan University; and (3) a diagnosis of LRTI. A patient was considered to have an LRTI if they had (1) at least one of the following manifestations of acute infection: confirmed fever (38 °C), abnormal white blood cell (WBC) differential, leukocytosis (a WBC count more than 10,000/mL) or leukopenia (a WBC count less than 4000/mL), and chills; (2) at least one of the following signs/symptoms of respiratory tract infection: cough, sputum, shortness of breath, lung auscultation abnormality (rale or wheeze), tachypnea, and chest pain.

The patients were divided into five groups: under 28 days of age (0–28 days), 1–12 months of age (> 1–12 months), 1–3 years of age (> 1–3 years), 4–6 years of age (4–6 years) and more than 7 years of age (> 7 years).

### Data and specimen collection

Respiratory specimens (nasopharyngeal aspirates/bronchoalveolar lavage fluid) were obtained from all the enrolled children within 72 h of admission by trained staff following standard operating procedures. The specimens were immediately transferred to the clinical laboratory for respiratory virus detection.

Demographics and clinical data from enrolled children were obtained from their electronic medical records.

### Specimen detection

A multiplex direct immunofluorescence assay (DFA) kit (Diagnostic Hybrids, Athens Ohio, USA) was used to detect respiratory viruses including respiratory syncytial virus (RSV), adenovirus (ADV), parainfluenza virus (PIV) 1–3, influenza virus A (FluA), influenza virus B (FluB) and human metapneumovirus (MPV) in the specimens. Additional quantitative real-time reverse-transcription polymerase chain reaction (qRT-PCR) testing was performed to detect rhinovirus (RV) using a commercial kit (Land Medical, Hubei, China) in accordance with the recommended protocol.

### Statistical analysis

Categorical variables were expressed as numbers (%). Continuous variables were expressed as medians (interquartile range). Medians for continuous variables (age, length of stay) between the year of 2020 and 2019 were compared using Mann–Whitney U test. Proportions for categorical variables (detection rates of virus, sex, intensive care unit admission, death in the hospital) were compared using the chi-square test. All of the tests were two tailed, and a value of *P* < 0.05 represented statistical significance. Statistical analyses were conducted in SPSS version 19.0 software (IBM, New York, USA).

## Results

### Study population

During the study period, 7107 patients who met the inclusion criteria were enrolled, including 4600 patients in 2019 and 2507 patients in 2020. The median age of patients in 2020 (2 months) was less than in 2019 (14 months), and the median length of hospital stay in 2020 (9 days) was longer than in 2019 (7 days). No significant differences in sex, intensive care unit admission, and death in hospital were observed between the year of 2019 and 2020 (Table 1).

**Table 1:** Characteristics and incidence of detected viruses in children with LRTI during the year of 2020 (COVID-19 pandemic) compared with the year of 2019 (before COVID-19 pandemic)

	2019 (n = 4600)	2020 (n = 2507)	*P* value
Characteristics			
Age, median (IQR), m	14 (1–48)	2 (0.5–12)	0.000
Male sex, n (%)	2685 (58)	1507 (60)	0.154
Length of stay, median (IQR), d	7 (4–13)	9 (6–16)	0.000
Intensive care unit admission, n (%)	1383 (30)	704 (28)	0.079
Death in the hospital, n (%)	68 (1.5)	26 (1.0)	0.120
Detection of viruses, n (%)			
RV	339 (7.37)	276 (11.01)	0.000
RSV	304 (6.61)	114 (4.55)	0.000
PIV3	177 (3.85)	54 (2.15)	0.000
ADV	135 (2.93)	4 (0.16)	0.000
FluA	60 (1.30)	9 (0.36)	0.000
PIV1	37 (0.80)	4 (0.16)	0.001
FluB	32 (0.70)	6 (0.24)	0.012
MPV	26 (0.57)	6 (0.24)	0.050
PIV2	9 (0.20)	1 (0.04)	0.179
Total	1072 (23.30)	460 (18.35)	0.000

### Overall detection of respiratory viruses

One or more viruses were detected in 460/2507 (18.35%) specimens in 2020, which was significantly lower than that in 2019 (1072/4600, 23.30%) (Table 1). RV was the most commonly detected virus, followed by RSV and PIV3, both in 2019 and 2020. The positive rate of RV was much higher in 2020 than that in 2019 (11.01% vs. 7.37%). However, compared with 2019, the positive rates of RSV, ADV, PIV1, PIV3, FluA, FluB, and MPV were significantly decreased in 2020. The positive rate of PIV2 had no significant difference between 2020 and 2019 (Table 1).

### Seasonal distribution

**Fig. 1:**
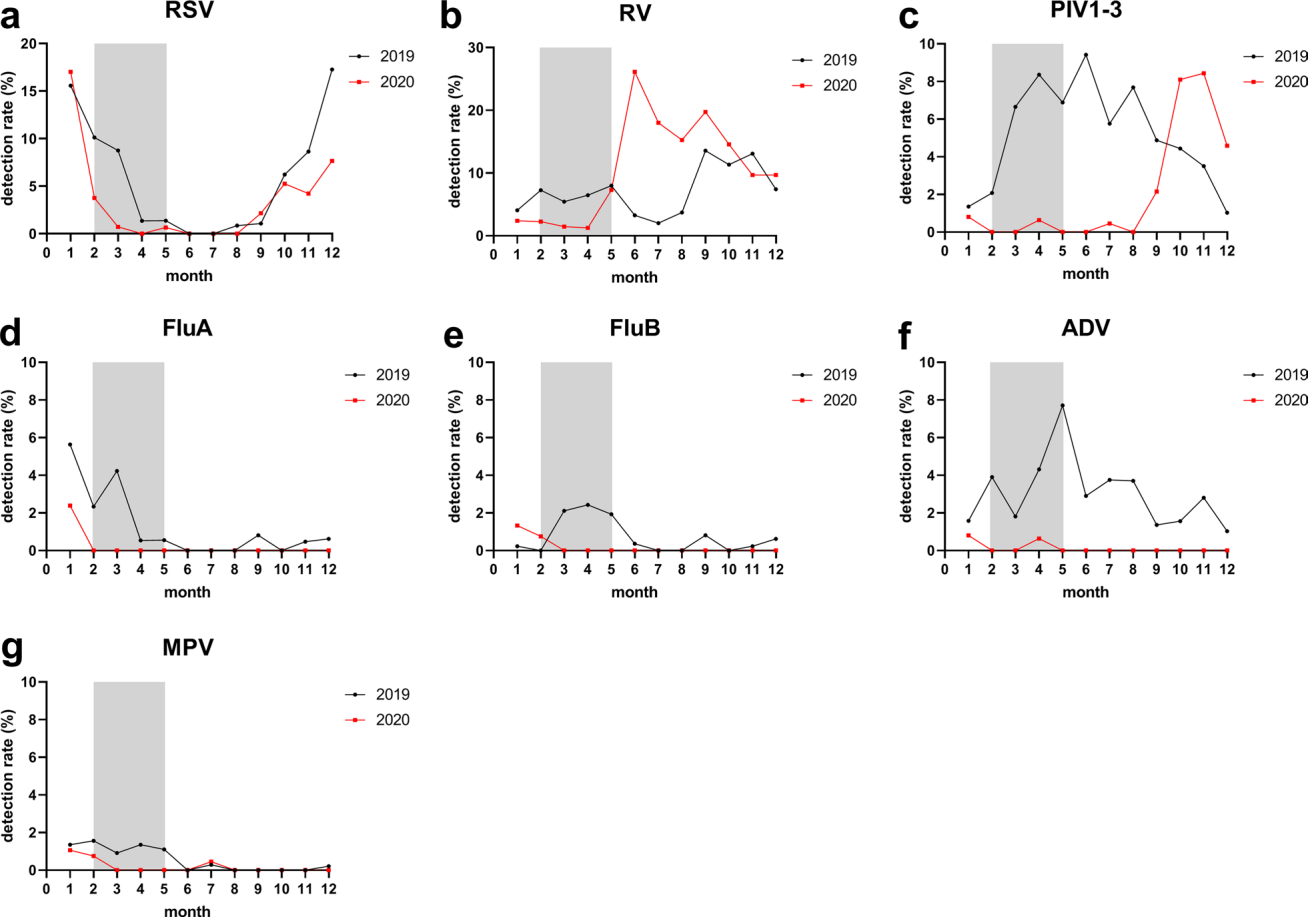
Seasonal activity of respiratory viruses during the COVID-19 pandemic year of 2020 (red line) compared with the previous year of 2019 (black line). Gray block represents the period from public health emergency of international concern (PHEIC) declared by World Health Organization (WHO) to the re-opening of primary and secondary schools

Compared with 2019, the detection rate of RSV decreased after February 2020 (WHO declared PHEIC). However, the seasonality did not change in 2020, and the detection rate was also peaked in winter (Fig. 1a).

RV was detected year round, with a high prevalence from June to October (after reopening of schools) in 2020, which was higher than the same period in 2019 (Fig. 1b).

PIV was almost not detected from March to August (spring and summer) in 2020, whereas it had a high prevalence in the same period in 2019. Interestingly, a sharp increase in the number of PIV detection was observed after August 2020 (Fig. 1c).

FluA, FluB, ADV and MPV were almost not detected after February 2020, and the low prevalence persisted even following the reopening of schools in early June. However, in 2019, FluA, FluB, and MPV had seasonal prevalence and ADV was prevalent throughout the year and peaked in May (Fig. 1d–g).

### Age distribution

All of the patients were grouped into five age groups.

**Fig. 2:**
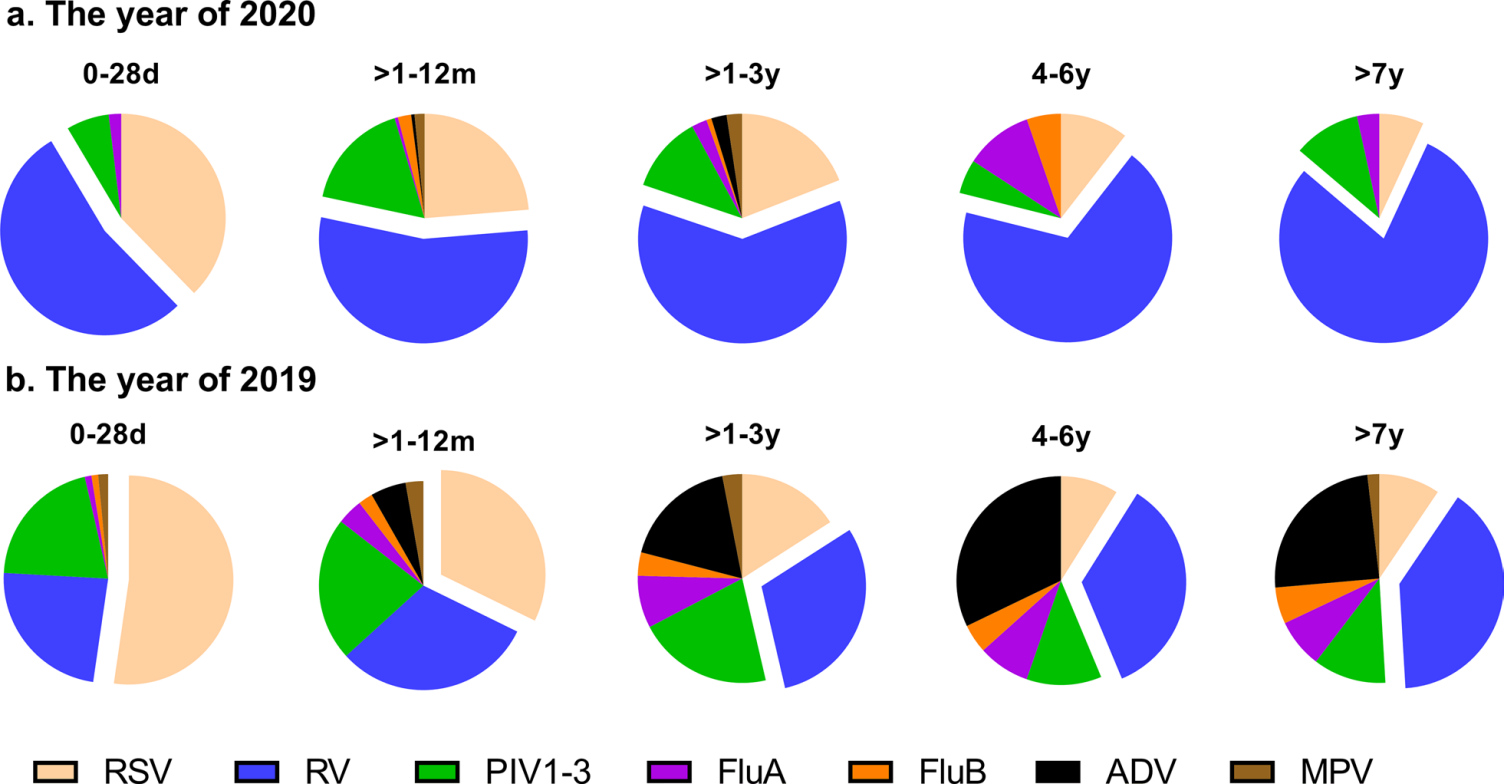
Proportions of respiratory viruses detected in 2020 (**a**) and 2019 (**b**) according to age group. The sliced part of each pie graph indicates the virus detected most frequently in that age group

The predominant viruses among different age groups between 2019 and 2020 varied. In 2019, RSV was the most frequent respiratory virus among children aged less than 12 months, and RV was the most common virus among children over 12 months of age (Fig. 2b). However, RV was the most frequent respiratory virus in all of the age groups in 2020 (Fig. 2a).

### Mixed viral infections

Mixed infections were significantly less frequent in 2020 (14/2507, 0.56%) than in 2019 (45/4600, 0.98%) (Table 2). RV was the most frequently found virus in mixed infections both in 2019 and 2020, accounting for 60.0% (27/45) and 92.9% (13/14) of all mixed infections respectively. Two triple infections were observed in 2019, whereas no triple infection was identified in 2020 (Table 2).

**Table 2:** Mixed infections of respiratory viruses in children with LRTI during the year of 2020 (COVID-19 pandemic) compared with the year of 2019 (before COVID-19 pandemic)

	2019 (n = 4600)	2020 (n = 2507)
Double infections, n (%)		
RV + PIV3	10 (0.22)	2 (0.08)
RV + RSV	6 (0.13)	9 (0.36)
RSV + PIV3	5 (0.11)	1 (0.04)
RV + PIV3	3 (0.07)	2 (0.08)
Other	19 (0.41)	0 (0.00)
Triple infections, n (%)		
RSV + PIV1 + ADV	1 (0.02)	0 (0.00)
RSV + PIV1 + RV	1 (0.02)	0 (0.00)
Total, n (%)	45 (0.98)	14 (0.56)

## Discussion

A series of strict NPIs adopted to contain the COVID-19 pandemic, including wearing masks, closing schools and social distancing, had not only decrease the spreading of the SARS-CoV-2, but changed the prevalence pattern of other common respiratory viruses [11]. In this study, we investigated the prevalence features of respiratory viruses in children with LRTI during the COVID-19 pandemic (the year of 2020) in Shanghai, China, and compared with the same date ranges in 2019. Our study showed a significant impact of COVID-19 pandemic on the circulation of common respiratory viruses in China.

In 2020, the number of children hospitalized with LRTI at Children’s Hospital of Fudan University was decreased by 45.5% compared with 2019. In accordance with our results, the significant decrease in the numbers of pediatric admissions in 2020 was also observed in many other countries, including France, Finland, United States, Brazil and Morocco [5, 12–15]. Avoidance of healthcare during the COVID-19 pandemic may be an important cause for the results presented. However, Matthijs et al. reported that a significant reduction was observed for children hospitalized with communicable infections (77%), whereas the reduction in noninfectious diagnoses was smaller (37%), which means the main reason for the reduction in pediatric admissions was the decrease in transmissible infections due to the strict interventions adopted in 2020, and avoidance of care could be a factor as well [16]. In other words, this reduction could be considered as an unexpected positive consequence of the NPIs taken during the COVID-19 pandemic, which may have led to a decrease in morbidity and healthcare costs of the infectious diseases.

Compared with 2019, the overall detection rate of respiratory viruses was lower in 2020 (18.35% vs. 23.30%). In another study conducted in New Zealand, the detection rates of influenza and other important respiratory viruses among inpatients with suspected severe acute respiratory illness (SARI) were also unprecedentedly reduced due to the use of stringent NPIs such as lockdown and border closures in 2020 [17].

Interestingly, in our study, the detection of RV appreciably increased, and become the most frequent respiratory virus in all of the age groups despite the NPIs adopted in 2020. However, there remains a drop in the detection rate of RV before the re-opening of primary and secondary schools in early June 2020, compared with the previous year. After the re-opening of school, there was a sharp increase in the number of detections, exceeding or being comparable to the same period in 2019. This resurgence of RV in 2020 was also reported in many other countries, such as Austria, New Zealand, United Kingdom and Japan [11, 17–19]. Since RV is a non-enveloped virus, it might be inherently less susceptible to inactivation by ethanol-containing disinfectant [20]. This viral property might hamper the prevention of RV infection using routine hand disinfectants. A previous study also demonstrated that surgical face masks could prevent the transmission of human coronaviruses and influenza viruses, but not of RV from symptomatic individuals with acute respiratory illness through respiratory droplets and aerosols [21]. Furthermore, virus–virus interactions can have impact on the incidence of respiratory virus infections, and FluA and RV have negative epidemiological interactions [22]. Thus, the increased detection of RV may result from the absence of FluA prevalence. Consequently, it is important that RV should continue to be monitored diligently in children during the COVID-19 pandemic.

Except for RV, the strict interventions adopted during the COVID-19 pandemic contribute to the significant reduction of all other respiratory viral infections, including RSV, PIV, FluA, FluB, MPV and ADV. Unlike the previous report from Australian, where the seasonality of RSV circulation has significantly changed and an increase in RSV detections occurred following the reduction of COVID-19 related public health measures [23], the seasonality of RSV in our study did not changed in 2020. Our previous study showed that PIV was detected more often in summer and had a low prevalence in winter [24]. However, there was a very low prevalence from January to August of 2020 but a sharp increase in the number of PIV detection after August. To the best of our knowledge, this resurgence of PIV has not reported in other studies. A study conducted in Japan demonstrated that frequency of PIV was appreciably reduced after the COVID-19 pandemic began, but their data was up to September 2020 [19]. Results for PIV suggested outbreaks may occur outside of the typical season during the COVID-19 pandemic. More broadly, these results also indicated that healthcare systems may need to prepare for future outbreaks of other common respiratory viruses, as COVID-19 restrictions are relaxed. However, the mechanism behind this resurgence is still unclear.

We have observed an almost absence of FluA, FluB, MPV and ADV prevalence since WHO declared PHEIC on January 31, 2020. The low prevalence of these viruses persisted even following the relaxation of COVID-19 restrictions in the latter part of 2020, which might attribute to the increased awareness of social distance, wearing face masks and hand hygiene. These findings also indicated that the NPIs had a remarkable effect on the prevention of the transmission of these viruses. Although the NPIs are not permanent, similar measures could be adopted to control future outbreaks of these respiratory viruses. Further, continuous surveillance and serological surveys are needed to better understand the long-term impact of the COVID-19 pandemic on the prevalence of common respiratory viruses.

## Conclusions

The NPIs during the COVID-19 pandemic have exerted a great impact on the prevalence of common respiratory viruses in China. An unprecedented reduction in influenza and other important respiratory virus infections were observed, most likely due to the implementation of NPIs in responds to COVID-19. Whereas, we do need to be cautious of a possible resurgence of some respiratory viruses as the COVID-19 restrictions are relaxed.

## Data Availability

Additional data are available from the first author and corresponding author upon reasonable request.

## Abbreviations

COVID-19: Coronavirus disease 2019
SARS-CoV-2: Severe acute respiratory syndrome coronavirus 2
NPIs: Non-pharmaceutical interventions
WHO: World Health Organization
PHEIC: Public health emergency of international concern
LRTI: Lower respiratory tract infections
RSV: Respiratory syncytial virus
RV: Rhinovirus
ADV: Adenovirus
PIV: Parainfluenza virus
FluA: Influenza virus A
FluB: Influenza virus B
MPV: Metapneumovirus
